# Co-Deletion of A238L and EP402R Genes from a Genotype IX African Swine Fever Virus Results in Partial Attenuation and Protection in Swine

**DOI:** 10.3390/v14092024

**Published:** 2022-09-13

**Authors:** Hussein M. Abkallo, Johanneke D. Hemmink, Bernard Oduor, Emmanuel M. Khazalwa, Nicholas Svitek, Nacyra Assad-Garcia, Jeremiah Khayumbi, Walter Fuchs, Sanjay Vashee, Lucilla Steinaa

**Affiliations:** 1 Animal and Human Health Program, International Livestock Research Institute (ILRI), P.O. Box 30709, Nairobi 00100, Kenya; 2 Department of Synthetic Biology and Bioenergy, J. Craig Venter Institute, Rockville, MD 20850, USA; 3 Institute of Molecular Virology and Cell Biology, Friedrich-Loeffler-Institut, Greifswald-Insel Riems, 17493 Greifswald, Germany

**Keywords:** ASFV, gene deletion, virulence, attenuation, protection, vaccine

## Abstract

African swine fever virus (ASFV) is the causative agent of African swine fever (ASF), resulting in up to 100% mortality in pigs. Although endemic in most sub-Saharan African countries, where all known ASFV genotypes have been reported, the disease has caused pandemics of significant economic impact in Eurasia, and no vaccines or therapeutics are available to date. In endeavors to develop live-attenuated vaccines against ASF, deletions of several of the ~170 ASFV genes have shown contrasting results depending on the genotype of the investigated ASFV. Here, we report the in vivo outcome of a single deletion of the A238L (5EL) gene and double deletions of A238L (5EL) and EP402R (CD2v) genes from the genome of a highly virulent genotype IX ASFV isolate. Domestic pigs were intramuscularly inoculated with (i) ASFV-Ke-ΔA238L to assess the safety of A238L deletion and (ii) ASFV-Ke-ΔEP402RΔA238L to investigate protection against challenge with the virulent wildtype ASFV-Ke virus. While A238L (5EL) gene deletion did not yield complete attenuation, co-deletion of A238L (5EL) and EP402R (CD2v) improved the safety profile of the single deletions, eliciting both humoral and cellular immune responses and conferred partial protection against challenge with the virulent wildtype ASFV-Ke virus.

## 1. Introduction

African swine fever (ASF) is a viral disease that results in up to 100% mortality in pigs depending on the genotype of the virus. The disease is endemic in most sub-Saharan African countries, where all the twenty-four African swine fever virus (ASFV) genotypes have been reported [1]. However, in Asia, Hispaniola, and Europe, where the disease currently causes dramatic losses, genotype II ASFV dominates. However, the emergence of genotype I ASFV in domestic pigs has recently been reported, particularly in the Henan and Shandong provinces of China [2]. Genotype I also remains prevalent in Sardinia (Italy).

The biological complexity of the virus and the dearth of knowledge about the mechanism of antiviral immune responses hampers the development of safe and efficacious vaccines. Despite decades of intense research, there is currently no commercial vaccine against ASF, although Vietnam has recently granted a commercial permit for local distribution of a live-attenuated ASF vaccine [3]. One approach employed to develop vaccine candidates against ASF is genetic attenuation of ASFV isolates through deletion of viral genes which are not essential for in vitro virus replication, and which are implicated in counteracting host immune responses. Some deletions have been shown to attenuate the virus and induce protection against homologous [4,5,6,7] and occasionally heterologous [8] challenges.

Among the numerous ASFV genes involved in evading host defence systems, A238L (5EL) encodes a protein with homology to IkB, the inhibitor of NFκB [9,10]. The A238L gene inhibits NFAT-regulated gene transcription in vivo and in vitro by inhibiting calcineurin phosphatase activity [11,12]. Since NFκB and NFAT pathways are involved in immune responses, their inhibition by A238L potentially suppresses the synthesis of pro-inflammatory cytokines. For instance, Salguero et al. demonstrated significantly enhanced expression of TNF-α mRNA in the PBMC from pigs inoculated with a ΔA238L virus, reinforcing the role of this gene in inhibiting the NFκB pathway of cytokine expression [13]. Similarly, Granja et al. presented evidence that A238L inhibits the activation of TNF-α by modulating NF-kB, NF-AT, and c-Jun transactivation [14]. Further, A238L inhibits the expression of the inflammatory regulators cyclooxygenase-2 (COX-2) [15,16] and TNF-α [16] and through inhibition of p65/RelA acetylation and p300 transactivation [17].

Additionally, a negative correlation between mRNA expression of the A238L gene and some cytokines has been observed in porcine macrophages infected with the highly-virulent L60 ASFV (ASFV/L60) [18], suggesting that the virulence of ASFV isolates may depend on their capacity to regulate the expression of the macrophage-derived cytokines relevant for the development of host protective responses. Deletion of the A238L gene in genotype IX ASFV does not alter the in vitro growth of the virus [19], as in genotype II Pol18/28298/Out111 [20] and genotype XII Malawi Lil-20/1 [21]. Furthermore, deletion of A238L in the highly virulent ASFV E-70 isolate does not result in attenuation, as no significant differences were observed in the clinical signs or pathology compared to the wildtype [13]. Similarly, growth characteristics in porcine macrophages and in vivo virulence phenotype (clinical outcome) of Malawi Lil-20/1-ΔA238L (5EL) were indistinguishable from those of the parental virus [21].

ASFV EP402R encodes CD2v, a protein with significant structural homology to the lymphocyte adhesion molecule CD2, which is required for the binding of erythrocytes to infected cells [22,23]. This was demonstrated by the hemadsorption of swine RBCs to Vero cells transiently expressing ASFV CD2v [23] and the loss of the hemadsorbing ability of the virus after genetic disruption of the EP402R gene [22]. Naturally occurring ASFV isolates with mutations or deletions in the EP402R gene have been shown to display a non-hemadsorbing phenotype [24,25,26,27,28,29,30]. Deletion of EP402R does not affect in vitro virus growth [19,22,31,32].

Deletion of the NL [33,34,35], 9GL [5,36], and UK [35,36,37] genes of ASFV suggest that the phenotypic outcome of deletion of specific virus genes is dependent on the genetic background of the parental virus. Deletion of the EP402R gene results in different phenotypic outcomes in vivo depending on the ASFV strain. However, deletion of the EP402R gene leads to complete attenuation of the virulent BA71 strain in pigs [8], the deletion of the same gene in ASFV Georgia 2010 [38], Malawi Lil-20/1 [31], and CN/GS/2018 [39] does not attenuate virulence in swine. Using the Kenyan genotype IX ASFV background, Hemmink et al. observed that deletion of EP402R resulted in reduced mortality and severity of clinical symptoms, albeit with retention of a degree of virulence [40].

To increase the safety profile of single deletion ASFV vaccine candidates, additional gene deletions have been included with diverse outcomes; some ASFV gene deletions like Δ9GL [5] and ΔMGF360/505 [36] confer protection on their own but lose the protective ability when co-deleted [36]. Conversely, simultaneous deletion of the 9GL and UK genes from the Georgia 2007 isolate offers increased safety and protection against homologous challenges [4].

In this study, we first examined whether the deletion of A238L in genotype IX ASFV-Kenya-1033 isolate (ASFV-Ke-IX-1033-∆A238L) [19] leads to a reduction in virulence upon infection of domestic pigs. Secondly, as the single deletion of the EP402R (CD2v) gene had already been tested [40], we assessed whether co-deletion A238L (5EL) and EP402R (CD2v) genes in the same virus (ASFV-Ke-IX-1033-∆EP402R∆A238L) [19] led to a reduction in virulence and whether immunity induced by this virus can protect against parental virus challenge.

## 2. Materials and Methods

### 2.1. Viruses

All the viruses used in this study have been reported previously, and their origins, the methodologies for generating them, their genomic sequences, and in vitro growth kinetics described [19,41,42,43,44].

Briefly, ASFV-Kenya-IX-1033, herein referred to as ASFV-Ke, is a genotype IX field isolate from the spleen of an infected domestic pig from the Busia district of western Kenya [41,42]. The isolate is highly virulent and genetically stable after passage in wild boar lung cell line (WSL) [44]. ASFV-Ke-ΔEP402R is a modified DsRed-expressing ASFV-Ke virus with the deletion of the EP402R (CD2v) gene [43]. ASFV-Ke-ΔA238L and ASFV-Ke-ΔEP402RΔA238L viruses are single- and double-deletion mutants, respectively, generated using the CRISPR/Cas9 approach [19].

### 2.2. Animal Experiments

Animal experiments were conducted in the ABSL2+ facility at the International Livestock Research Institute (ILRI, Nairobi, Kenya). Pigs, which were crosses between Duroc and Large White, weighing between 45 and 70 kg, were ear-tagged, weighed, dewormed, and vaccinated for foot and mouth disease (FMD) and held in a quarantine facility for at least twenty-one days and acclimatized for seven more days prior to the start of the experiment. As FMD is endemic in the region, it is standard practice to vaccinate susceptible species when they arrive in the quarantine unit. Only healthy animals, as determined by clinical examinations, seronegative for ASFV, and negative by p72 qPCR, were included in the study. For the attenuation experiment (IACUC2021-10), two groups of three pigs were each inoculated intramuscularly with 10^2^ TCID_50_ of ASFV-Ke-ΔA238L or 10^2^ HAD_50_ ASFV-Ke and observed for 21 days to assess the safety of the deletion mutant. For the protection experiment (IACUC2020-11), two groups of nine pigs were each inoculated intramuscularly with 10^3^ TCID_50_ dose of ASFV-Ke-ΔEP402RΔA238L or PBS. Eight pigs per group were challenged intramuscularly on day 31 with 10^2^ HAD_50_ of wild type ASFV-Ke. Baseline samples were collected prior to inoculation on day 0 and subsequently at periodic intervals; days 3, 5, 7, 10, 14, and 21 post-inoculation for the IACUC2021-10, days 3, 7, 14, 21, and 28 post-inoculation, and days 3, 5, 7, 10, 14, 21 post-challenge for IACUC2020-11. These were used to determine in vivo viral loads and antibody levels at different time points. Heparinized blood was collected on day 28 to determine the immune responses of the different animal cohorts to the viruses used for vaccination compared to controls. Animals were monitored daily for clinical signs and scored according to King et al. [45]. Animals were euthanized when pre-determined humane end-point criteria were reached or at the end of the study.

### 2.3. PBMC Isolation

Peripheral blood mononuclear cells (PBMCs) were isolated from 20 mL of blood collected in heparinized vacutainer tubes (Ref: 368480, BD) using the Ficoll-Paque^TM^ (GE Healthcare, Uppsala, Sweden) density gradient centrifugation. Briefly, heparinized blood was mixed with an equal volume of sterile PBS, layered on Ficoll-Paque, and centrifuged at 1650× *g* for 30 min at room temperature with no brakes. The interphase layer containing PBMCs was harvested, washed with sterile 1 × PBS, and spun at 670× *g* for 10 min with brakes on. The pellet was resuspended in 10 mL of tris-ammonium chloride and incubated in a water bath set at 37 °C for 10 min to lyse residual RBCs. The cell suspension was spun at 300× *g* for 10 min to remove residual platelets. The cell pellet was washed twice by suspending in 1 × PBS and spun at 300× *g* for 10 min. Isolated PBMCs were 40 µm-filtered to remove fat and resuspended in 20 mL of RPMI medium supplemented with 10% fetal bovine serum (Ref: A4766, GIBCO), 1% L-glutamine (Ref: 21051040, Thermo Fisher Scientific, Waltham, MA, USA), and 1% Penicillin/Streptomycin (Ref: HP10.1, Roth). PBMCs were counted in a Neubauer chamber using the Trypan Blue dye exclusion method.

### 2.4. Porcine IFNγ ELISPOT

ELISPOT plates (Millipore, Ref MAHAS4510) were coated overnight at 40 °C with 100 µL per well of mouse anti-pig IFNγ clone P2G10 capture antibody (Ref: 559961, BD Pharmingen) diluted 1:1000 in 0.2 µm-filtered carbonate buffer. The plates were washed five times using sterile 1 × PBS (200 µL per well) and blocked with 4% skimmed milk (Marvel, Premier Foods group, Thame, UK) for 2 h at room temperature. The plates were washed five times with PBS before adding 50 µL of antigens; Concanavalin A (10 ug/mL), wildtype ASFV-Ke (MOI = 0.1. Five hundred thousand PBMCs from each animal were seeded in the respective wells and plates were incubated for 20 h at 37 °C with 5% CO_2_.

Plates were developed by washing five times with 200 µL per well of PBS-Tween (0.05% Tween-20 diluted in 1 × PBS). Biotinylated mouse anti-pig IFNγ primary antibody (100 µL), clone P2C11 (BD Pharmingen) diluted 1:2000 in PBS was added per well and incubated for 2 h at room temperature. Plates were washed five times using PBS-Tween, before adding 100µL of the secondary antibody, streptavidin alkaline phosphatase (Invitrogen), diluted 1:1000 in PBS. After 1 h incubation at room temperature, the plates were then washed in PBS-Tween. Thereafter, 50 µL of the SIGMAFASTTM BCIP^®^/NBT substrate (Ref: B5655, Sigma) was added per well and incubated for 20 min in the dark. The plates were washed with tap water to stop the reaction and then immersed in 1% formaldehyde solution for 10 min to inactivate residual viruses. Finally, the plates were washed in tap water and kept in the dark to air dry. The spots were counted using the AID classic ELISPOT reader (AID AutoImmun Diagnostika GmbH. Results were reported as spot-forming units (SFU) per 1 × 10^6^ PBMC.

### 2.5. Porcine Anti-p72 ELISA

Five millilitres of blood were collected from each animal in serum separating vacutainer tubes (BD) and centrifuged at 1200× *g* for 10 min to separate serum. ASFV anti-p72 antibodies were detected by blocking ELISA using the INgezim PPA Compac Kit (INGENASA, Madrid, Spain) as per the manufacturer’s instructions. The optical densities of the developed plates were read immediately at 450 nm wavelength using the Synergy HTX multi-mode reader (Ref: S12FA, BioTeK, Winooski, VT, USA). The quality control analysis and percent blocking were determined according to the manufacturer’s protocol. Samples were considered positive if the percentage blocking was above the 50% blocking cut-off value.

### 2.6. Real-Time Quantitative PCR for the Detection of ASFV Genome

Viremia in experimentally inoculated animals was quantified at different days post-infection using quantitative PCR (qPCR). Genomic DNA was extracted from 200 μL of EDTA anti-coagulated blood using a Zymo Quick-DNA miniprep DNA extraction kit (Ref: D3025, Zymo research, Irvine, CA, USA). For the detection of ASFV genome copies in tissues, approximately 0.01 g to 0.025 g of splenic, submandibular, and gastrohepatic lymph tissue was weighed and DNA extracted using the Qiagen DNeasy Blood and Tissue Kits (Cat# 69506, Qiagen, Hilden, Germany). qPCR was performed using QuantStudio^TM^ 5 system (Applied Biosystems, Waltham, MA, United States). Each reaction was conducted in duplicates in a 10 μL reaction mixture containing 2.23 μL nuclease-free water, 5 μL EXPRESS qPCR Supermix (Invitrogen, Waltham, MA, USA), 0.3 μL of forward primer (10 μM), 0.3 μL of reverse primer (10 μM), 0.15 μL of TaqMan^®^ probe (10 μM), 0.02 μL of ROX reference dye, and 2 μL template DNA. The plasmid standard dilutions, primers, and qPCR conditions are described by Abkallo et al. [19]. Data, in eds file format, were exported and analysed on a QuantStudio™ design and analysis software (Applied Biosystems, United States). Results were analysed on GraphPad Prism (version 6) for Windows (GraphPad Software, San Diego, CA, USA).

## 3. Results

### 3.1. Safety of ASFV-Ke-∆A238L in Pigs

**Figure 1:**
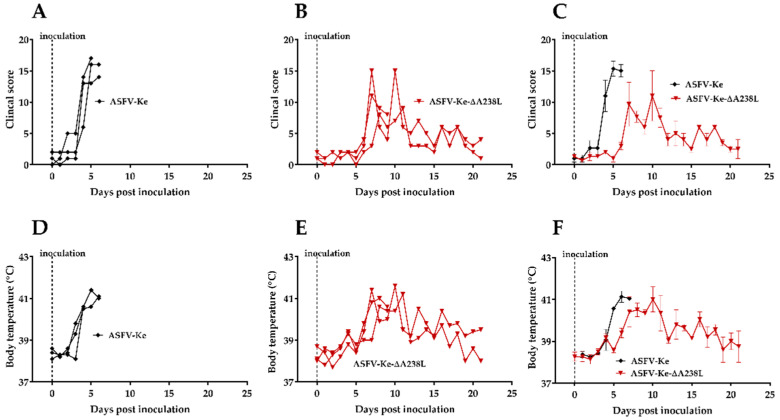
Body temperature and clinical scores: Rectal temperatures in individual pigs inoculated with 10^2^ HAD_50_ of ASFV-Ke (**A**) or 10^2^ TCID_50_ ASFV-Ke-∆A238L (**B**). Mean +/− SEM of rectal temperatures in ASFV-Ke (black) and ASFV-Ke-∆A238L (maroon) groups (**C**). Cumulative clinical scores in pigs inoculated with ASFV-Ke (**D**) or ASFV-Ke-∆A238L (**E**). Mean +/− SEM of cumulative clinical scores in ASFV-Ke (black) and ASFV-Ke-∆A238L (maroon) groups (**F**).

**Figure 2:**
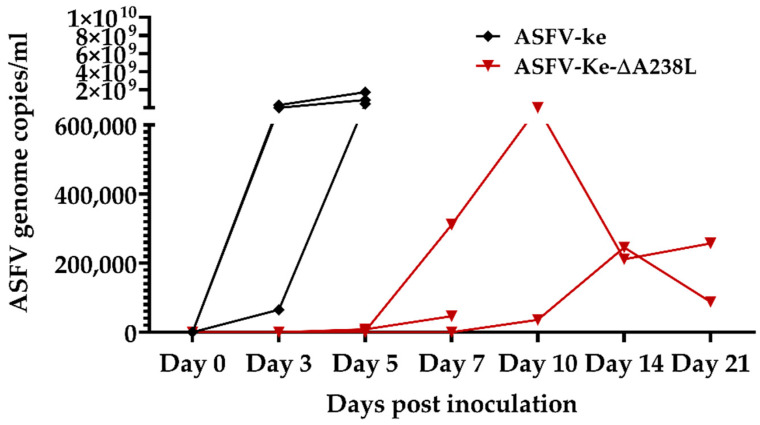
Virus replication in the blood (viral genome copies) after inoculation with 10^2^ TCID_50_ of ASFV-Ke (black) or ASFV-Ke-∆A238L (maroon).

To assess the safety of ASFV-Ke-∆A238L, two groups of three pigs were each intramuscularly inoculated with either the parental wildtype virus ASFV-Ke or the A238L gene-deleted mutant (ASFV-Ke-∆A238L), and were observed for 21 days or until humane end-point criteria were reached. The pigs in the ASFV-Ke wildtype control group all developed fever (>40 °C) and clinical signs typical of ASF and were euthanised 5- or 6-days post-inoculation (Figure 1A and Figure S1). In contrast, two out of three pigs of the ASFV-Ke-∆A238L group survived the experiment (Figure 1B and Figure S1A). The non-surviving pig in the ASFV-Ke-∆A238L group was euthanised on day 9 post-inoculation. High amounts (up to 1.7 × 10^9^ copies/mL) of viral DNA were detected in the blood of all ASFV-Ke group members until day 5 pi, while much lower copy numbers were detected in ASFV-Ke-∆A238L group (Figure 2). Additionally, there was a delay in the increase in viral genome copies in the ASFV-Ke-∆A238L group (Figure 2), probably due to slower replication of the deletion mutant, or the ability of the innate host immune response to suppress virus growth. The body temperature in the ASFV-Ke-∆A238L group increased above the 40 °C threshold on day 7 post-inoculation and declined gradually until normalising (≤40 °C) on day 14 and 17 post-inoculation (Figure 1B). On average, body temperature increased more rapidly, and clinical scores were much higher in the ASFV-Ke group than in the ASFV-Ke-A238L group (Figure 1C,F). Together, these observations indicate that the ASFV-Ke virus is highly virulent in pigs, whereas ASFV-Ke-∆A238L is moderately attenuated.

### 3.2. Induction of ASFV-Specific Humoral Response in ASFV-Ke-∆A238L-Inoculated Pigs

**Figure 3:**
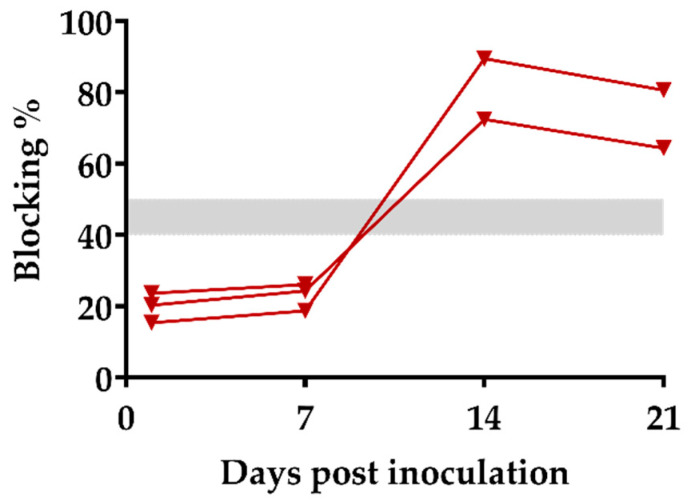
ASFV-specific antibody response after inoculation with ASFV-Ke-∆A238L. The percentage of blocking by p72-specific serum antibodies at indicated times was tested using a commercial competitive ELISA. The threshold range is indicated by a grey bar.

Antibody responses to ASFV-Ke-∆A238L virus were measured using a commercial competitive ELISA in sera obtained at different days post-inoculation. From day 14 post-inoculation, samples from the two surviving pigs showed 89.5% and 72.4% blocking, which is above the 50% positivity threshold (Figure 3). The antibody response was maintained until the end of the observation period (day 21 post-inoculation), illustrating induction of ASFV-specific humoral immune response by the ASFV-Ke-∆A238L virus.

### 3.3. Safety and Protective Efficacy of ASFV-Ke-∆EP402R∆A238L Double Deletion Mutant

To assess the safety and immunogenicity of the double deletion mutant (ASFV-Ke-∆EP402R∆A238L), a group of nine pigs were intramuscularly (i.m.) immunised with 10^3^ TCID_50_ dose of the ASFV-Ke-∆EP402R∆A238L virus. In parallel, another group of nine pigs were mock-immunised with PBS (control). Both groups were observed for 30 days until challenge i.m. with 10^2^ HAD_50_ of the virulent wildtype ASFV-Ke.

**Figure 4:**
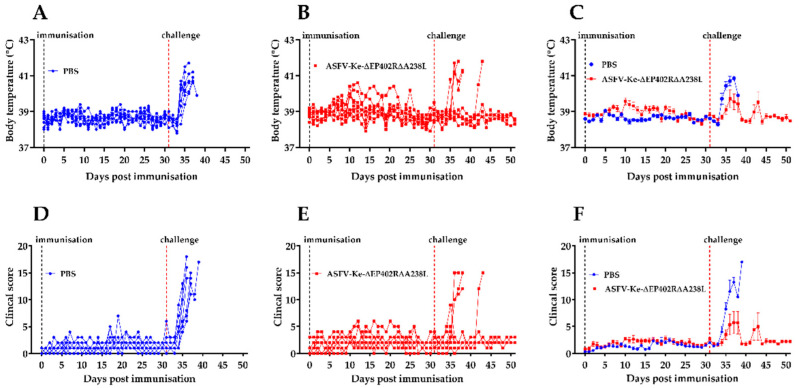
Body temperatures and clinical scores: Rectal temperatures in individual pigs mock-immunised with PBS (**A**) or immunised with 10^2^ TCID_50_ of ASFV-Ke-∆EP402R∆A238L (**B**) before and after challenge with ASFV-Ke. Mean +/− SEM of rectal temperatures in PBS (blue) and ASFV-Ke-∆EP402R∆A238L (red) (**C**) and ASFV-Ke challenged groups. Cumulative clinical scores in pigs mock-immunised with PBS (**D**) or ASFV-Ke-∆EP402R∆A238L-immunised (**E**) and ASFV-Ke challenged groups. Mean +/− SEM of cumulative clinical scores in mock-immunised (blue) and ASFV-Ke-∆EP402R∆A238L-immunised (red) (**F**) and ASFV-Ke challenged groups.

**Figure 5:**
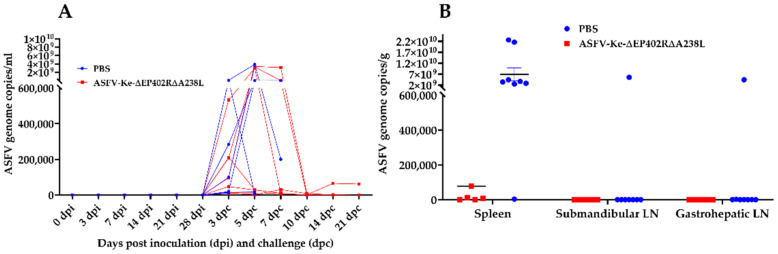
Virus replication in the blood (viral genome copies) after immunisation with PBS (blue) or ASFV-Ke-∆EP402R∆A238L (red) and subsequent challenge with ASFV-Ke (**A**). Detection of virus DNA in tissues prepared post-mortem from animals immunised with PBS (blue) or ASFV-Ke-∆EP402R∆A238L (red) after challenge with ASFV-Ke (**B**).

After immunisation, the majority of the ASFV-Ke-∆EP402R∆A238L-immunised pigs maintained normal temperature (seven out of nine pigs) and low clinical scores (all pigs) during the 30-day follow-up period (Figure 4B,E), indicating that the ASFV-Ke-∆EP402R∆A238L double deletion mutant is safe, and attenuated to a greater degree than the single mutants. No viral DNA was detected in blood of eight pigs, and one pig had viral genome copies below the threshold value (Figure 5), indicating that the pigs were able to limit viral replication in vivo.

Prior to challenge infection, one randomly selected animal was removed from each group to meet the approved animal number of eight. After challenge with the virulent wildtype ASFV-Ke, fifty percent of the ASFV-Ke-∆EP402R∆A238L-immunised pigs survived the study duration, while the other 50% reached their humane end-point criteria on days 6, 7, and 12 post-challenge (Figure S1B). Five pigs in the ASFV-Ke-∆EP402R∆A238L-immunised group exhibited an increase in body temperature and developed clinical signs consistent with ASF (Figure 4B,E). However, three pigs in the same group maintained normal body temperature, and no clinical signs were observed (Figure 4B,E). All control (PBS-immunised) pigs were euthanised by day 7 post-challenge after reaching the humane end-point (Figure 4A,D and Figure S1B). Higher copy numbers of viral DNA were detected in both groups by day 3 post-challenge (Figure 5). However, by day 10 post-challenge, viral DNA copies declined to zero in three of the four surviving pigs in the ASFV-Ke-∆EP402R∆A238L-immunised group (Figure 5A). On average, clinical scores and temperatures in the ASFV-Ke-∆EP402R∆A238L-immunised group were much lower compared to the mock-immunised group. ASFV DNA was undetectable in both the submandibular and gastrohepatic lymph nodes and low in the spleen tissues of ASFV-Ke-∆EP402R∆A238L-immunised pigs (Figure 5B). In contrast, ASFV genome copy numbers were much higher in the spleens of mock-immunised pigs (Figure 5B).

### 3.4. Induction of ASFV-Specific Humoral and Cellular Responses in ASFV-Ke-∆EP402R∆A238L-Immunised Group

**Figure 6:**
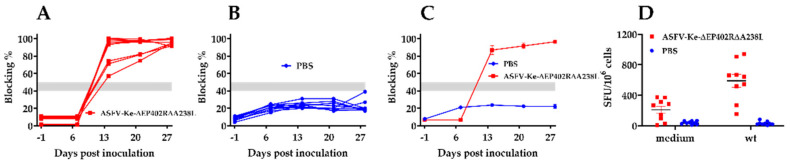
ASFV-specific antibody and cellular immune responses: percentage of blocking by p72-specific serum antibodies after mock-immunisation with PBS (**A**) or immunisation with ASFV-Ke-∆EP402R∆A238L (**B**) determined using a commercial blocking ELISA. Mean+/−SEM of percentage blocking in PBS- or ASFV-Ke-∆EP402R∆A238L-immunised groups (**C**). IFNγ ELISPOT using PBMC isolated from animals at day 28 after immunisation with PBS (blue) or ASFV-Ke-∆EP402R∆A238L (red) and stimulation with either medium or ASFV-Ke wild type (wt) infection at an MOI of 0.1 (**D**).

Analysis of ASFV-specific antibodies to ASFV-Ke-∆EP402R∆A238L was performed using a commercial competitive ELISA. Antibody responses were detected and firmly established in all animals by day 14 post-immunisation, with 100% blocking seen in six of nine pigs (Figure 6B). Antibody titres were maintained in all pigs throughout the experiment, demonstrating that ASFV-Ke-∆EP402R∆A238L immunisation induced ASFV-specific humoral responses.

To investigate the induction of ASFV-specific cellular immune response in pigs immunised with ASFV-Ke-∆EP402R∆A238L, an ELISpot assay was used to measure the number of cells producing IFN-γ. ASFV-specific IFN-γ-producing cells (up to ~940 spots per million cells) were detected in PBMC of seven of the ASFV-Ke-∆EP402R∆A238L-immunised pigs (Figure 6D). The remaining two pigs in the ASFV-Ke-∆EP402R∆A238L-immunised group did not have detectable ASF-specific IFN-γ responses. No ASFV-specific IFN-γ responses were found in mock-immunised animals. The ELISA and ELISpot results show that ASFV-Ke-∆EP402R∆A238L virus is immunogenic.

## 4. Discussion

In studies of ASFV gene function, and in order to generate live-attenuated vaccines (LAV) against ASF, targeted disruption or deletion of viral genes is a standard practice [4,5,6,7,31,36,38,46,47]. Previous studies have led to different attenuation and protection results depending on the genotype and virulence of the virus isolates studied [8,31,38,39,46,47]. An ideal LAV should have an acceptable safety profile (reduced or absent clinical signs) and be able to provoke robust protection against challenge infection with wild type ASFV. To evaluate whether deletion of A238L in genotype IX ASFV reduces virulence compared to the parental strain, pigs were inoculated with the wild type ASFV-Ke or ASFV-Ke-∆A238L and monitored for clinical manifestation of infection. Although both groups developed clinical signs consistent with ASF, clinical scores were, on average, much higher in the ASFV-Ke group compared to the ASFV-Ke-∆A238L group.

Additionally, the deletion of the A238L gene from the genotype IX ASFV-Ke delayed the onset of clinical signs by three days in the infected pigs. In line with this, there was a substantial decrease and delay in the detection of ASFV DNA in the blood of ASFV-Ke-∆A238L-inoculated pigs. This observation reveals partial attenuation of the ASFV-Ke-∆A238L mutant, and differs from observations in ASFV E-70 and ASFV Malawi Lil-20/1 isolates, in which A238L deletion had no significant phenotypic outcome in vitro or in vivo [13,21]. Reduced clinical scores and reduced rate of viral multiplication in pigs inoculated with the ASFV-Ke-∆A238L may also point to enhanced innate immune responses against the mutant virus, which supports the proposed immune evasion functions of A238L [13].

Since single deletions of the EP402R gene [40] and A238L did not independently yield complete attenuation in ASFV-Ke, we investigated the attenuation and protection profiles of an A238L and EP402R double deletion mutant. Compared to ASFV-Ke-∆A238L-inoculated pigs, ASFV-Ke-∆EP402R∆A238L-inoculated pigs maintained normal temperature, exhibited mild clinical scores, and were able to limit ASFV replication. These observations suggest that the ASFV-Ke-∆EP402R∆A238L double deletion mutant is safer than the ASFV-Ke-∆A238L single deletion mutant. However, comparing this ∆EP402R∆A238L double deletion with the ∆EP402R single deletion [40], the double deletion is not superior to the ∆EP402R single deletion.

Upon being challenged with the highly virulent wildtype ASFV-Ke virus, three out of eight (37.5%) of the ASFV-Ke-∆EP402R∆A238L-immunised pigs maintained normal temperature and developed no clinical signs besides limiting viral replication in the blood. Additionally, ASFV genome copy numbers were significantly lower in the spleens of ASFV-Ke-∆EP402R∆A238L-immunised pigs than in the mock-immunised control pigs, showing that immunised pigs can inhibit virus replication efficiently. However, only half of the pigs were sufficiently protected against a lethal challenge which was less than obtained with ASFV-Ke-∆EP402R single deletion mutant, where only one out of eight animals was lost [40]. Thus, additional deletion of A238L apparently reduces the protective efficacy of the EP402R (CD2v) single knockout virus. Similar phenomena have been described for the ASFV Georgia strain where the addition of a CD2v deletion and/or a C-type lectin-like deletion to a 9GL deleted virus abrogated protection [46].

In conclusion, the single knockout ASFV-Ke-∆A238L was not sufficiently attenuated to develop as a vaccine on its own, and the double knockout ASFV-Ke-∆EP402R∆A238L, while sufficiently attenuated, did not confer adequate protection. These results demonstrate the delicate balance between attenuation and protection observed in gene-deleted ASFV viruses. However, these problems could possibly be mitigated by higher vaccine doses or booster injections, although this may not be optimal in resource-constrained settings, such as sub-Saharan Africa.

## Data Availability

Not applicable.

## Supplementary Materials

The following supporting information can be downloaded at: https://www.mdpi.com/article/10.3390/v14092024/s1, Figure S1: Kaplan-Meier survival curve of ASFV-Ke (black)- or ASFV-Ke-∆A238L (maroon)-inoculated pigs (A), and PBS (blue)- or ASFV-Ke-∆EP402R∆A238L (red)-immunised pigs after challenge with wildtype ASFV-Ke (B).
