# Supplementary figures and images for: Co-Deletion of A238L and EP402R Genes from a Genotype IX African Swine Fever Virus Results in Partial Attenuation and Protection in Swine
Source: Viruses. 2022 Sep 13;14(9):2024. doi: 10.3390/v14092024 (PMC9501025; doi:10.3390/v14092024)

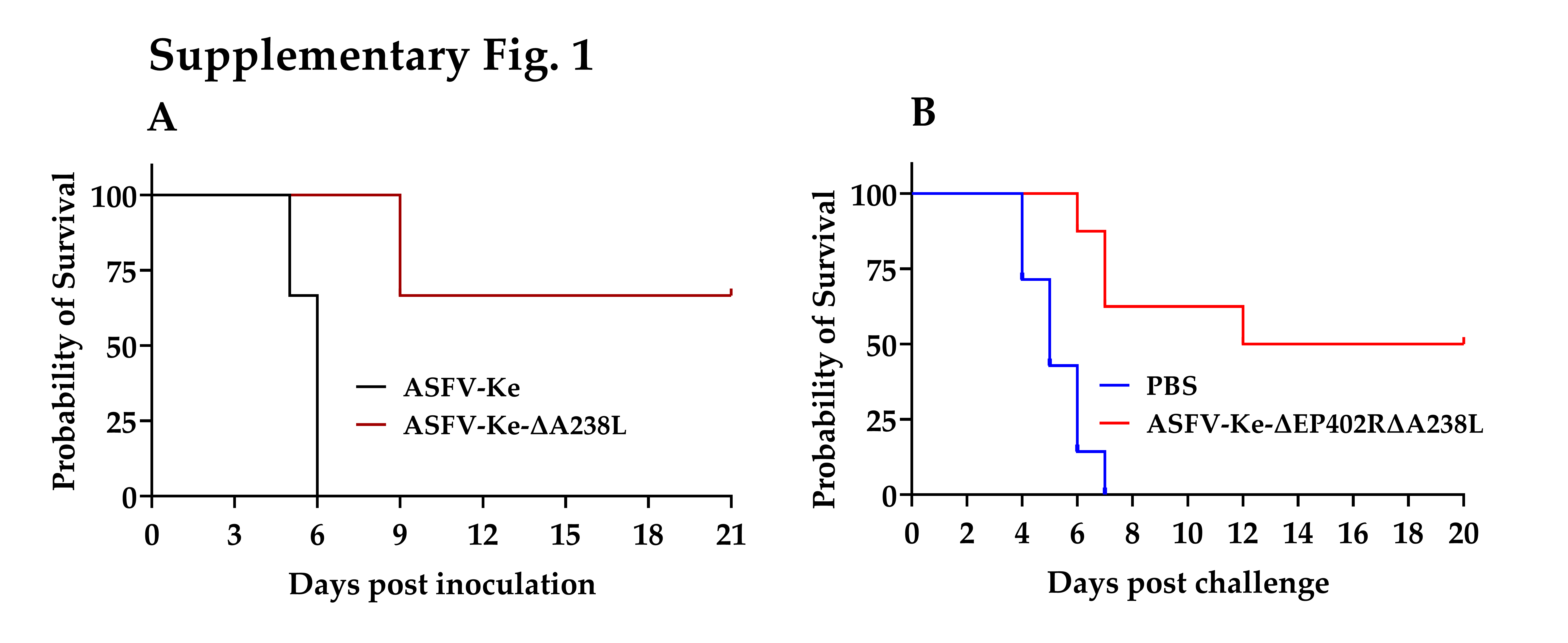

Supplement: Supplementary file 1 [file viruses-14-02024-s001.zip › Supplementary Fig 1_rev.tif]
